# Contracting in specialists for emergency obstetric care- does it work in rural India?

**DOI:** 10.1186/1472-6963-12-485

**Published:** 2012-12-31

**Authors:** Bharat Randive, Sarika Chaturvedi, Nerges Mistry

**Affiliations:** 1 Department of Public Health and Environment, R D Gardi Medical College, Ujjain, India; 2 Formerly with Foundation for Research in Community Health(FRCH), Pune, India; 3 Ph D scholar at Karolinska Institutet, Stockholm, Sweden; 4 Foundation for Research in Community Health, Pune, India

**Keywords:** Contracting in, Public private partnership, Emergency obstetric care, India, Janani Suraksha Yojana

## Abstract

**Background:**

Contracting in private sector is promoted in developing countries facing human resources shortages as a challenge to reduce maternal mortality. This study explored provision, practice, performance, barriers to execution and views about contracting in specialists for emergency obstetric care (EmOC) in rural India.

**Methods:**

Facility survey was conducted in all secondary and tertiary public health facilities (44) in three heterogeneous districts in Maharashtra state of India. Interviews (42) were conducted with programme managers and district and block level officials and with public and private EmOC specialists. Locations of private obstetricians in the study districts were identified and mapped.

**Results:**

Two schemes, namely Janani Suraksha Yojana and Indian Public Health standards (IPHS) provided for contracting in EmOC specialists. The IPHS provision was chosen for use mainly due to greater sum for contracting in (US $ 30/service episode vs.300 US$/month). The positions of EmOC specialists were vacant in 83% of all facilities that hence had a potential for contracting in EmOC specialists. Private specialists were contracted in at 20% such facilities. The contracting in of specialists did not greatly increase EmOC service outputs at facilities, except in facilities with determined leadership. Contracting in specialists was useful for non emergency conditions, but not for obstetric emergencies. The contracts were more of a relational nature with poor monitoring structures. Inadequate infrastructure, longer distance to private specialists, insufficient financial provision for contracting in, and poor management capacities were barriers to effective implementation of contracting in. Dependency on the private sector was a concern among public partners while the private partners viewed contracting in as an opportunity to gain experience and credibility.

**Conclusions:**

Density and geographic distribution of private specialists are important influencing factors in determining feasibility and use of contracting in for EmOC. Local circumstances dictate balance between introduction or expansion of contracts with private sector and strengthening public provisions and that neither of these disregard the need to strengthen public systems. Sustainability of contracting in arrangements, their effect on increasing coverage of EmOC services in rural areas and overlapping provisions for contracting in EmOC specialists are issues for future consideration.

## Background

India’s slow progress in reducing maternal mortality not only hinders its own goals but also affects the global achievement of MDG 5 as India accounts for about one fourth of the maternal deaths worldwide [1]. The five most common causes of maternal mortality in India are direct obstetric complications which can be well managed by provision of emergency obstetric care (EmOC) services. The Indian government subscribed to the EmOC provision strategy and in 2005 included access to 24 hour EmOC as one of the ‘service guarantees’ of its public health programme-the National Rural Health Mission (NRHM). The mission launched a safe motherhood programme titled Janani Suraksha Yojana (JSY) which provides cash incentives to mothers conditional to their delivering in health institutions [2]. The programme logic is increased access to institutional care during delivery would decrease delays in accessing EmOC services if required and thus save lives.

Another challenge in India is health workforce shortages and difficulties in recruiting and retaining them especially in rural and remote areas as in most low and middle income countries (LMICs). The Indian census data shows health worker density about half of the WHO benchmark of 25.4 such workers associated with achieving 80% institutional deliveries attended by skilled personnel in cross country comparisons [3]. The Indian public health system suffers from a severe shortage of specialists to deliver EmOC- 37% positions of obstetricians are vacant and moreover compared to existing requirements there is a shortfall of 55% obstetricians in rural India [4]. Contrastingly, the private sector share in services has grown significantly, despite the high expenses involved.

The NRHM also set the Indian Public Health Standards (IPHS) [5] for service provision. Along with other improvements in the infrastructure, achieving these standards would require a large increase in numbers and skills of human resources [6]. Thus India sees a phenomenal increase in the number of interventions and strategies towards this, some specific to states while others nationwide. Some alternatives being explored are task shifting for anaesthesia and obstetric services, decentralising recruitment to district level and altered processes like holding walk in interviews, posting junior residents at CHCs for fixed period, providing financial and career prospect incentives [6]. The extent of success of each initiative to address skilled personnel shortages needs to be viewed against the contexts that influence them. For example some initiatives face legal issues with licensing of providers raised through alternate arrangements, performance rewards, career pathways and job clarity [7]. However as noted by a recent overview of systematic reviews there are few studies of the potential of the various initiatives and their effect on the size and distribution of EmOC providers and the related service uptake, specifically from LMICs [8].

With NRHM's strategy to promote public private partnerships, there have been numerous initiatives in various areas in health care; however caution is suggested in execution of these to achieve efficiency and equity [9]. A recent popular public private partnership scheme focusing on EmOC in India is the Chiranjeevi Yojana [10] in Gujarat state which is a contracting out model with voucher based financing, whereas other states have contracting in. The private sector though large is concentrated in urban areas. Also feasibility of fruitful partnerships with unregulated private sector is questioned, for instance a study of private maternity homes in Maharashtra state signals possible compromise in service quality with poor standards in private sector [11].

The NRHM promotes contracting in of specialists for EmOC services and has made financial provisions for the same under two of its prominent initiatives – the JSY and the IPHS. The JSY is the world’s largest conditional cash transfer (CCT) programme in health in terms of number of beneficiaries. While public spending on JSY has been increasing since its launch in 2005-06 [12] a steep rise in institutional births from 40.7% in 2005-06 [13] to 68% in 2008-09 [14] has been concomitantly observed. However studies do not associate this spurt of institutional deliveries with a decrease in maternal mortality [15].

While the JSY has been studied widely, to the best of our knowledge, there is no report specifically on the contracting in component for EmOC provision in the context of the JSY as also of the IPHS. This paper attempts to fill this gap by reporting on the implementation of contracting in and its effect on EmOC availability in an Indian state. The findings should provide insights to policy makers on the design and implementation of contracting in for EmOC in resource constrained settings.

## Methods

This was an exploratory study. A mix of quantitative and qualitative methods was used including a facility based cross sectional survey and provider interviews.

### Study settings

This study was conducted in Maharashtra state in western part of India. It has a higher level of economic growth compared to the national average, however in terms of Human Development Index (HDI) that reflects the social determinants of health; it ranked eleventh in 2006 with a HDI value of 0.689. Maharashtra has 35 districts comprising 358 blocks. The state has better health indicators than the national; maternal mortality ratio is 104 and infant mortality rate is 31 against 212 and 50 for India respectively. The public health system in the state is three tiered as in the rest of the country having tertiary care centres at districts, secondary care centres at sub district/block level and primary health care centres within blocks. Maharashtra has highest number of private medical colleges in the country and health care sector is dominated by private providers.

### Selection of study districts

Three districts were selected on the basis of heterogeneity in their socio-demographic profiles as reflected in Human Development Report Maharashtra 2002 [16] and assuring state wide geographic representation. The study districts thus selected were Nandurbar (low HDI), Amravati (Moderate HDI) and Satara (better HDI) using the latest data available at the time of selection. While Nandurbar is a tribal district with high Schedule Tribe (ST) population, Amravati has a high population of poor people i.e. those living Below the Poverty Line (BPL) and belonging to Scheduled Castes (SC). On the other hand, Satara has a relatively high uptake for institutional births (Table 1).

**Table 1:** Socio demographic indicators of study districts

**Districts**	**Population**					**Institutional deliveries**		*HDI Rank
	Total	Urban(%)	*SC(%)	*ST (%)	*BPL(%)	Rural (%)	Total (%)	
Satara	28,08,994	14.2	9.5	34.7	43	85.6	87.4	10
Amravati	26,07,160	34.5	17.5	14.4	66	50.5	63.6	15
Nandurbar	13,11,709	15.4	3.2	65.5	54.5	16.5	25.3	32
Maharashtra	9,68,78,627	42.4	10.2	8.8	30.7	54	63.6	

Source: Census 2001, DLHS – 3, Human Development Report Maharashtra 2002.

*SC-Schedule Caste ST- Schedule Tribe BPL- Below Poverty Line HDI- Human Development Index.

### Selection of health facilities

We studied all the public secondary and tertiary care centres i.e. district hospitals (DH), sub district hospitals (SDH) and community health centres (CHCs) in the selected three districts. Thus 44 health facilities in the study districts- 3 DHs (250 bedded), 8 SDHs (50- 100 bedded), and 33 CHCs (30 bedded) were included. (Table 2) All the study centres are expected to provide Comprehensive EmOC (CEmOC).

**Table 2:** Public health facilities surveyed

	**District hospitals**	**Sub district hospitals**	**Community health centres**	**Total**
Satara	1	2	15	18
Amravati	1	4	9	14
Nandurbar	1	2	9	12
Total	3	8	33	44

### Survey of health facilities

We surveyed each health facility using a modified health facility assessment form based on the facility survey for EmOC designed by the UN agencies and the Averting Maternal Death and Disability programme [17]. Information on availability of skilled manpower for provisioning of EmOC services was obtained and detailed information was gathered on implementation of contracting in model for private EmOC specialist. Also data on performance of obstetric services by facility in six months prior to survey were retrieved from records at facilities. Study team members with medical training conducted the survey. The data collection at health facilities was undertaken intermittently during December 09 to August 2010. Each facility was visited once without prior notice. In this paper we report data specific to contracting in for EmOC.

### Interviews with providers and managers

Interviews were conducted with private EmOC specialists (n=15) either contacted in or located in vicinity of facilities with potential for contacting in and with medical superintendents and public EmOC specialists (n= 20) at selected facilities. Interviews were also conducted with district health officials, and district and block programme managers (n=7).

### Mapping of obstetricians

We obtained a list of all practising private obstetricians in the three study districts from the district hospitals. We confirmed this list with practising obstetricians and also used snowballing to complete it. We also approached members of the professional association of obstetricians and gynaecologists at the study districts for validation of the list.

### Data collection procedures

Informed written consent was obtained from all study participants. All interviews were audio taped and transcribed verbatim for analysis.

### Data analysis

Quantitative data was entered and analysed in Excel. Qualitative data was analysed using thematic content analysis using deductive coding.

### State support

We obtained formal bureaucratic support for the study from the State Health Systems Resource Centre, National Rural Health Mission (NRHM) Division, Government of Maharashtra.

### Ethics approval

Approval for the study was granted by the Institutional Ethics Committee of the Foundation for Research in Community Health, Pune,India.

## Results

### 1. Provision for contracting in

The provision of contracting in private specialists for EmOC services is made under two initiatives of the NRHM:

a. JSY: The JSY provides a fixed amount of INR 1500 (~ US$30) to be paid to a contracted in EmOC specialist for every complicated delivery attended.

b. IPHS: Broad guidelines are issued by state authorities and funds for contracting in specialists are made available to facilities. The guidelines mention that the facility management committee can decide on the amount to be paid for services contracted. We found that the range is set at district level and amount in Amravati and Satara district was fixed INR 20,000 (~400 USD) per specialist per month.

### 2. Potential for contracting in

#### Specialist shortage in rural public facilities

Of the 41 facilities at sub district level, 18 had obstetrician(s) while 10 had an anesthetist. However the number of facilities that had both these specialists was 7 (17%) which implies that there were 34 facilities (83%) that had a potential for undertaking contracting in for EmOC specialists with variations within districts as seen in Table 3.

**Table 3:** Availability of emergency obstetric care specialists at rural public facilities

**District**	**Facility (n)**	**Ob/Gyn & Anaes**	**Ob/Gyn. only**	**Anesth only**	**Neither**
Amravati	13	0	1	0	12
Satara	17	4	8	3	2
Nandurbar	11	3	2	0	6
Total	41	7	11	3	20
Total (%)		7 (17%)	34 (83%)		

#### Specialist concentration in private sector

The findings revealed a high concentration of obstetricians in the private sector. Viewing the number of obstetricians in the private sector against the population of the districts (Census 2011) we arrived at the concentration of obstetricians in the private sector as presented in Table 4. There was an obstetrician per 243000 populations in the public sector while 1 per 29000 in the private sector with much variation across districts - increased concentration proportional to development of the district. Overall there were about eight times more obstetricians in the private sector than in the public sector (Table 4).

**Table 4:** Availability of obstetricians in study districts

**District**	Govt. Ob/popln*	Pvt. Ob/popln*	Total Ob/popln*
Amravati	1/3,60,978	1/26,016	1/24,267
Satara	1/2,00,261	1/23,107	1/20,716
Nandurbar	1/2,05,772	1/86,640	1/60,969
Total	1/2,43,158	1/28,992	1/25,903

### 3. Practice of contracting in EmOC specialists

#### No contracting in under JSY

We found that private specialists had not been contracted in under the JSY at any of the facilities studied. There had been no initiative to contract in specialists under the JSY and most of the medical superintendents were not aware specifically that the JSY had a provision for contracting in for EmOC. They were aware of contracting in through IPHS and of the JSY provision as meant for subsidisation of Caesarean section (C section) cost at private facilities.

*The JSY guidelines are clear that whatever the benefit, it has to be given to those patients who come and who deliver or have C section, it can be given only to the patients, you cannot involve the doctors in that*. *Whatever hiring has to be done for speciality services of doctors, can be done under the IPHS.* - Medical Superintendent

The scheme however appeared functional since the funds allocated for it were being utilised in alternative ways mentioned below.

#### JSY provision for contracting in specialist used as a subsidy for C section

The most frequently reported use of the JSY provision (for contracting in specialist) was to provide this amount to JSY eligible mothers undergoing C section at private facilities turning it into Government subsidy (partial) against C section costs at private hospital. The JSY guideline mentions of this as an alternative mechanism to offer a choice of place of delivery to mothers and provide for areas where contracting in has not been done. It provides for paying INR 1500 (approx 30 $) to the specialist who provides services to JSY eligible mothers in a private facility.

#### Contracting in under IPHS

Contracting in private EmOC specialists was undertaken as part of facility strengthening for the IPHS at 7 (20%) of the 34 facilities that had vacant positions of EmOC specialists. Ten facilities with such potential did not have functional operation theatre and hence did not attempt contracting in for EmOC. Procedures for contracting in under IPHS were uniform across districts. The executive body of the Rugna Kalyan Samiti (RKS) (a facility level management body formed after the NRHM comprising of local community members and medical superintendent as its president) was given the powers and authority to administer and manage the contracts with private providers. Specific posts are advertised in local news papers and applications received are scrutinised by the medical superintendent who then proposes names of candidates to the executive committee. The committee then interviews the candidates for final selection. The Resident Medical Officer (RMO outreach) of the district represents the civil surgeon on the committee and guides the medical superintendent on executing the contract.

Our interviews revealed that although there were established procedures, it was mostly the leadership and relations of medical superintendents with private specialists that primarily influenced the execution of contracts. The following quote elaborates this:

*it is more of personal rapport with private doctors, some of them are very cooperative. And this is two way, much depends on how we approach them, our personal relations with them matter, also age factor, if they are of the same age group or sometimes they are classmates, so these things matter-* Block Medical Officer

As regards the selection process, we learnt from our interviews that it remained only a formality to be completed as choices were often limited. Wherever choices existed, preference was given to the nearest specialist.

*What to select, actually there is no much choice here*- District official

*. the first preference is that the person should be a local, because he is easily available, one who has to travel may not give good services. Of the local doctors (specialists), we call for interview, one who accepts the lower salary, we appoint that person* - District official

We found that the contract design was poor with respect to the terms and conditions specified in the contract.

*The specialist hired gets an appointment letter saying hired as gynaecologist at such a facility for such a period; it’s signed by the authority. It’s not like a contract signed by two parties, it’s just like an appointment letter*. – District official

The contracts with obstetricians generally included two hours of outpatients on alternate days in a week and taking emergency calls as and when required. These contracts with obstetricians were for a fixed amount about INR15-20,000 (~300-400 USD) per month while those with anaesthetists were on an event basis, about INR 800-1000 (~200 USD) for a C section or any major operation and INR 500 (10USD) for minor cases. The contract duration extended generally for eleven months.

The contract conditions however did not determine the nature of service delivery. Here too the specialists’ rapport with the medical superintendent influenced actual service delivery rather than the contract terms.

*We give them a call once a few patients (for outpatients) have gathered, they also have their private practice, they come and see those, then after some time when few more patients are gathered, we again call them so they make two such rounds. This all is going just for mutual adjustments.* - Medical Superintendent

*They are private specialists, they come here only after they finish their own work, but we are friends so we manage. -* Medical Superintendent

There was no orientation or training at any level in the public system about contracting in. One medical superintendent recollected a mention of this during an orientation to the NRHM. The rest recollected that it was conveyed during various routine meetings at district level.

Additionally there were no mechanisms for monitoring performance of private specialists serving in CHCs under such contracts. Most officials and medical superintendents opined that any stringency with the private partner would be counterproductive for patients’ accessibility to services. One of them said

*If we are too strict of the timings, we will not get people. If it is a diabetic or hypertensive case, she might have to wait for some time, but she at least gets the services, rather than nothing. –* Medical Superintendent

It was also mentioned that private specialists were habituated to prescribing certain drugs that are not available in CHCs.

*if he writes from what is available here, patients get that here, but he writes as he would be writing in his practice, there are many newer drugs that we do not have, those are prescribed from outside.* – Medical Superintendent

The renewal rate of contracts was noted to be high, at over 80-85%. There were no contracts terminated by the authorities in any of the districts.

*Termination …it is not from our side, mostly if they themselves want to leave, only then we do not renew it (contract)…. –* District official

### 4. Performance of contracting in

We found overall nine of the 41 sub district level facilities provided C section services.

#### Influence of contracting-in on service output

A comparison of specific service outputs (C sections) at facilities with contracting in specialists with those with regular staff (no contracting in) is presented in Table 5. Although other EmOC services are equally important, we chose to compare C section for availability of data and specificity to contracting in, as C section is currently performed only by specialists.

**Table 5:** C-sections at facilities with and without contracting in specialist for EmOC

**Facilities with contracting in specialists**	**Nu of C sections (April-Sept 09)**
1	78
2	62
3	1
4	6
5	6
6	4
7	0
Facilities with no contracting in (government specialists)	
1	20
2	2

The number of C sections at most facilities, either with contracted in specialists or with government specialists was noted to be quite low for a six month period. The relatively better performance of the two facilities with contracted in specialists (Table 5) points to leadership issues at specific facilities. It was noted that the medical superintendents at these facilities were keenly interested in increasing the availability of C section services at these hospitals. These findings show that, with a few exceptions, overall contracting in has hardly increased the availability of C section at rural public facilities.

#### Influence of contracting in on emergency services

It was noted that the contracting in arrangement was not useful for emergencies. It was being used primarily for elective cases. This was mostly attributed to the distance to private specialists contracted in making it unfeasible to provide their services in emergency conditions. Also doctors select cases for elective surgeries at rural centres avoiding chances of complications as quoted below:

*Because we select the patient properly and we take good (non risky) patients there, I have told the surgeon, if you want to do it there, you keep good patients, keep fit patients only, that’s why there has been no complication in the periphery.*- Contracted in anaesthetist

The contracted in specialists prefer providing services in the private sector due to relatively larger gains than in lending their service to public hospitals. This was another reason for the contracts to be used only for elective cases.

*..No anaesthetist is willing to come in morning hours, they come here only in afternoons, , because they have their own link in morning hours with the private hospitals, that might break (if they come here in mornings) , they have their link for the whole week, what will they do by taking one day from me, that’s why I start my OT at 2 (in the afternoon) .. .So I do not think it (hiring) is much useful in emergencies but definitely useful for elective and planned cases.* – Medical Superintendent

### 5. Barriers to contracting in

#### Logistics barriers

The main reasons for not contracting in were that the facilities did not have the required infrastructure for EmOC provision.

Availability of blood storage facilities was very limited: all district hospitals had blood storage facility but at sub district level only 10% facilities were so equipped. Of the facilities at sub district level, 41% did not have a functional operation theatre, 88% stocked injection Oxytocin while only 34% stocked Injection Magnesium Sulphate (Table 6).

**Table 6:** Infrastructure for EmOC provision at rural public facilities in study districts

**Rural public facilities n=41**	**Labor room**	**Functional OT**	**Lab**	**Blood storage**	**Power to OT 24x7**	**Water**	**USG**	**Ambulance**	**Drugs**		**Specialist Accomodation**
									**MgSo4**	**Oxytocin**	
% age of availability	95	59	88	10	80	95	15	78	34	88	54

OT- Operating Theatre USG- Ultra Sonography MgSO4-Magnesium Sulphate.

#### Private provider availability in vicinity

Contracting in for EmOC was influenced by the unavailability of private specialist near to the CHC with such a potential. The distance to the nearest private specialist for 50% of the needy facilities was noted to be more than 30 Kms.

It was noted that the seven facilities where contracting in was done were those where the private specialist was located in the same town or within 20 km distance as seen in figure-1 below (Pl see Additional file 1).

Also owing to the mountainous terrain and poor quality roads in Nandurbar district, the travel time was relatively extended. This probably affected the specialists’ willingness to be contracted in and the feasibility of the arrangement.

*The problem is it takes 1 hour and a quarter more to reach here from (names town), though the distance is not much, but the area is hilly. So for a private specialist there this means 2.5 hours of travel time plus work time here, which would not match with their own practice, so they are willing to come only once or twice a week.-* Medical Superintendent, (Please see figure provided in Additional file 1)

We did not find any utilization of the JSY provision for contracting in mainly because the JSY amount is lower than the usual charges for such services. As the financial provision for contracting in under IPHS was much higher than in the JSY, district officials preferred to execute the contracts under the IPHS.

### 6. Contracting in strategy- views and experiences

#### Public partners’ views

Government officials expressed different opinions about the contracting in strategy and its utility to the public system. There were specific comments about contracting in as a strategy and of the arrangements for it in the JSY.

Overall, it was felt that the strategy could be of immediate use, but not as a solution to the shortage of specialists in the system.

*. it's useful that way. Otherwise we cannot give these services (EmOC) if there are no government specialists. It is essential until government has own full time specialists. How can MBBS doctors do all that*? – District official

Some senior officials expressed that given peoples preference to private providers, involving them in service delivery at public facilities may help in changing people’s perceptions about public hospitals.

*The patients like that they are being seen by a private specialist who would charge a consulting fee of INR 150 outside, they are getting it done free here* – Medical Superintendent

*It’s a good scheme, useful to the public system. The fact is village people’s opinion of private sector is good, so if they are coming in our facilities, it will definitely have a positive effect on us. –* Block Medical Officer

The officials who did not believe that the PPP scheme would be of much use explained why they held this opinion. One of them indicated the need to go to the root cause of the shortage in the system while another elaborated on perspectives of private providers doubting that this arrangement could be overtly fruitful.

*In Government service, medical officers must be on regular basis only, there should be no need to get specialists this way (contract from private), why does such a need arise should be thought of by the Government*. – District official

The government specialists interviewed mentioned how the government is spending on paying the private specialists while neglecting the ones who are in service.

*.now you are giving it (money) to private doctors, there are also specialists in the government, pay them also; why would one not go and do a Caesarean in the nearby CHC if this 1500-2000 (INR) that is given as incentive to private doctors is given to us* – Government Obstetrician

The arrangement for contracting in as in the JSY was not appealing to the administrators either who felt that it does not ensure sustained services, one official explains as

*See, the fact is that, at a hospital where gynaecologist does not come regularly, a patient coming just for a Caesarean or any surgery is rare, means the patient is not willing to get it done there , that’s not utilised very effectively- that’s why (the contracting in under JSY) has not grown that well. But what we have done under IPHS is working well because they are doing it all over the month*. – District official

The contracting in arrangement was also seen as an easy way of getting patients to private hospitals. Senior officials tended to agree with the above opinion as one of them elaborated

*One thing is they like patients to be referred to their own hospital, any private hospital owner would want that obviously, and other thing is they have so many sophisticated equipments at their hospital, which are not available at the CHC level; even if they are in the IPHS, they will take time to upgrade, so it is so safe and secure to work there (*own set up*),so they ask to send patients to their hospitals.* - District official

#### Private providers’ experiences and views of the scheme

The private specialists viewed the contracting in as an opportunity to gain clinical experience.

*…It is both monetary gains and the experience of working in government set up…-* Private obstetrician

*…here we keep only usual cases, but it’s mainly the bulk experience that counts, doing more and more number of cases,so.*- Contracted in obstetrician

The contracting in arrangement is seen as a good platform for beginners.

*… I came here in February 2008, till then I was studying, my husband is practising here since three years, so I wanted to start to create my base here, initially I had no set up and no work to do, so I joined here, now my private practise is also good*- Contracted in obstetrician

The private specialists preferred to work in their own or other private set ups. An anesthetist explained her preference to calls for anesthesia from private hospitals

*.means my calls from the private are regular calls whereas from the government there are not so many, that’s why I would obviously first take the private call.* – Contracted in anaesthetist

A senior obstetrician who was recently approached for contracting in differed from his younger colleagues:

*No,not so, because I am already half way through my career and I do not want my benefits any more. If it is benefiting the patients, well and good. –* Private obstetrician

Experiences of private specialists working through the contracting in arrangement were of dissatisfaction about the availability of infrastructure and practices in public hospitals.

*And their OTs are also not good, not properly sterilised, so many patients get infection there.* – Private obstetrician

*…even I was a bit,.(hesitant) giving anaesthesia without Boyles machine and resuscitation equipment, although spinal, …then I used to carry my own resuscitation set. –* Private anaesthetist

## Discussion

This being the very first study to our best knowledge that reports on issues of contracting in specialists in the context of the renewed focus on EmOC in India, it is exploratory in nature. The exploratory nature of our study and its focus on rural areas need to be considered while interpreting the findings. Also it needs to be kept in mind that contracting in is an evolving and dynamic process. We discuss the findings in three sub sections below.

### Contracting in for EmOC in rural contexts

Although existing resource constraints make pooling of capabilities and resources necessary, the strategic choice of contracting in specialists seems rational only in selected regions- for instance in regions characterised by shortages of public and concentration of private specialists, as in two of the districts in this study while it proves unfeasible in the other district. This indicates that no single measure could be applicable to all regions and that area specific issues are important influencing factors. There need to be locally relevant strategies to provide EmOC services. A district like Satara with relatively higher concentration and density of private specialists offers more opportunities for contracting in and broad bargaining zone that could be explored by engaging in dialogue with the private sector while another district like Nandurbar in the state has a dearth of specialists even in private sector rendering contracting in unfeasible. For instance of regional variations in strategies the Gujarat experiment of harnessing private sector capacity through the Chiranjeevi scheme which is by contracting out services is greatly divergent from the Tamil Nadu experience with strengthening human resources within the Indian system and by contracting in providers [18]. However the root cause- the shortages of specialists in public service and the long term sustainability of such arrangements needs to be considered. Our findings suggest that local circumstances will dictate balance between introduction or expansion of contracts with private sector and strengthening public provisions and that neither of these disregard the need to improve public systems. These findings corroborate conclusions from a multi country study by Mills [19] and additionally suggest that these hold for sub national levels too. A review of literature on contracting with the private sector for primary care in LMICs concludes that theories from new institutional economics and evidence about nature of existing contracts for primary care in the United Kingdom question the viability of a policy of competitive contracting in in the context of LMICs [20]. Also alternative arrangements like task shifting that involves delegation of tasks to lower level cadre have shown promising results in the developing world [21]. For instance the post operative outcomes of C sections performed by non physician providers in Malawi were comparable to those of doctors [22]. However the only available ,though early, evaluations of task shifting for EmOC-for both C section and anaesthesia in India are not encouraging and demand greater support [23,24]. The choice revolves around what is the most effective way to improve coverage, reach and quality of EmOC services and reduce its costs in the specific contexts. Also it is important to recognise that contracting in for EmOC is additionally complex than for other clinical and diagnostic services, which is gaining popularity in developing countries like India, as time to response is crucial in saving lives in the former.

### Public provisioning for contracting in

The overlapping provision for contracting in under JSY and under the IPHS points to the isolation of policy making from implementation channels. There appear to be multiple provisions for a poor woman eligible for the JSY requiring EmOC- while IPHS spends to provide these services, the JSY allocates to subsidise her costs in private hospitals in addition to routine outlays for functioning of public health facilities. However despite these measures the spending and bankruptcy of poor households to access these services continue.

In planning a public health programme care needs to be taken to ensure resources are not duplicated. This is not to deny the need for interventions but to make the right choice for the larger good. The Accountability for Reasonableness Framework advocated by Mitton and Donaldson et al. [25] recommends four conditions that could be used to test a public spending decision. These are publicity of the rationale for spending, relevance of the rationale to the context of the decision, appeals to allow feedbacks and change and enforcement of the decision made. The bottom line is whether the public spending decisions are driven by economics and ethics. There is currently no measure of the marginal gains of one scheme over the other. Use of administrative and managerial tools like Programme Budgeting and Marginal Analysis, also termed as options appraisal, which also provides for inclusion of users perspectives in programme management are highly recommended. Also application of concepts of allocative efficiency that addresses the issue of achieving the right mixture of healthcare programmes to maximise the health of society are essential.

### Management of contracting in arrangements

Contracting in is within itself a skill demanding job when it comes to implementation at grass roots. The ignorance to developing the required skills in the public system is not a good sign especially when contracting in is seen as panacea to all specialist shortages in the system. Our findings are in agreement with those from authors of other studies on PPP in health in India [26] in that they emphasise the need to develop capacities in public system personnel in designing and managing contracts. In the absence of experience in developing and administering contract instruments, as our findings show especially with recent decentralisation of this authority to lower levels, there ought to be mechanisms to innovate and evaluate. Also as concerns have been raised of the ability of private providers to serve for public interests, designing mechanisms for overcoming challenges to mutual trust, logistical arrangements and financial transactions like social auditing [27] could be beneficial.

## Authors’ contribution

All authors contributed to the conceptualisation and design of the study. BR and SC participated in the data collection and analysis. BR wrote the first draft of this paper. SC and NM contributed to revise the draft and BR wrote the final version. All authors read and approved the final version. The authors declare that they have no competing interests.

## Prior dissemination

A part of the data presented in this paper was shared at the National Conference on Bringing Evidence to Public Health Policy, organised by Institute of Tropical Medicine, Belgium and Institute of Public Health, Banglore, India, December 2010. The abstract of this conference is published in BMC *Proceedings*, 6 (Suppl 1):01 (16 January 2012).

## Pre-publication history

The pre-publication history for this paper can be accessed here:

http://www.biomedcentral.com/1472-6963/12/485/prepub

## Supplementary Material

Additional File 1: Figure S1 showing distance to private EmOC specialists (figure location- findings section under subheading 5.2). Click here for file
