# Supplementary material for: Contracting in specialists for emergency obstetric care- does it work in rural India?
Source: BMC Health Serv Res. 2012 Dec 31;12:485. doi: 10.1186/1472-6963-12-485 (PMC3572412; doi:10.1186/1472-6963-12-485)
Supplement: Additional File 1: Figure S1 — showing distance to private EmOC specialists (figure location- findings section under subheading 5.2). [file 1472-6963-12-485-S1.doc]

**0**

**10**

**20**

**30**

**40**

**50**

**60**

**70**

**80**

**90**

**100**

**110**

**1**

**2**

**3**

**4**

**5**

**6**

**7**

**8**

**9**

**10**

**11**

**12**

**13**

**14**

**15**

**16**

**17**

**18**

**19**

**20**

**21**

**22**

**23**

**24**

**25**

**26**

**27**

**28**

**29**

**30**

**31**

**32**

**33**

**34**

**Amravati**

**Satara**

**Nandurbar**

**Distance (km)**

**Obstetrician**

**Anaesthetist**

**Districts**

Fig-1: Distance of private EmOC specialists from Community Health Centres
